# Cannabis use and opioid relapse: An exploratory survival analysis of prospectively collected data

**DOI:** 10.3389/fpsyt.2022.1046649

**Published:** 2022-11-16

**Authors:** Leen Naji, Tea Rosic, Nitika Sanger, Brittany Dennis, Alannah Hillmer, Jacqueline Hudson, Andrew Worster, James Paul, David C. Marsh, Lehana Thabane, Zainab Samaan

**Affiliations:** ^1^Department of Family Medicine, McMaster University, Hamilton, ON, Canada; ^2^Department of Health Research Methods, Evidence, and Impact, McMaster University, Hamilton, ON, Canada; ^3^Department of Psychiatry, University of Ottawa, Ottawa, ON, Canada; ^4^Medical Sciences Graduate Program, McMaster University, Hamilton, ON, Canada; ^5^Department of Medicine, McMaster University, Hamilton, ON, Canada; ^6^Neuroscience Graduate Program, Department of Psychiatry and Behavioural Neurosciences, McMaster University, Hamilton, ON, Canada; ^7^Department of Psychiatry and Behavioral Neurosciences, McMaster University, Hamilton, ON, Canada; ^8^Department of Anesthesia, McMaster University, Hamilton, ON, Canada; ^9^Northern Ontario School of Medicine, Laurentian University, Sudbury, ON, Canada; ^10^Biostatistics Unit, Research Institute at St Joseph’s Healthcare, Hamilton, ON, Canada

**Keywords:** cannabis use, opioid use disorder, relapse, buprenorphine, opioid agonist therapy

## Abstract

**Importance:**

It is known that only minority of patients with opioid use disorder (OUD) receive treatment, of which only a fraction successfully complete treatment as intended. Factors associated with poor treatment outcomes remain unclear, and there is emerging but conflicting evidence that cannabis use may mitigate opioid use.

**Objective:**

To analyze predictors of relapse amongst patients receiving buprenorphine-naloxone for OUD and identify the association between cannabis use and time to relapse.

**Design:**

Data were prospectively collected between May 2018 and October 2020, and patients were followed for 12 months.

**Setting:**

Thirty-one outpatient opioid agonist treatment clinics across Ontario, Canada.

**Participants:**

All patients 16 years of age or older receiving buprenorphine-naloxone for OUD who had a urine toxicology screen negative for opioids at baseline were eligible for inclusion. Of the 488 patients consecutively sampled, 466 were included.

**Exposure:**

Cannabis use.

**Main outcome and measure:**

Relapse to opioid use assessed using urine toxicology screens. We employed a multivariable Cox-proportional hazard model for our analyses.

**Results:**

We found that cannabis use was not protective against relapse [hazard ratio (HR) = 1.03, 95% confidence interval (CI): 0.78, 1.36, *p* = 0.84]. We found that participants who have been in treatment for at least two years had a 44% decrease in the hazard of relapse compared to those in treatment for less than a year (HR = 0.56, 95% CI: 0.34, 0.92, *p* = 0.021). We also found that the hazard of relapse was 2.6 times higher for participants who were intravenous drug users (HR = 2.61, 95% CI: 1.74, 3.91, *p* < 0.001), and that for every 1mg increase in the participants’ buprenorphine-naloxone dose, the hazard of relapse is 2% greater (HR = 1.02, 95% CI: 1.01, 1.03, *p* < 0.001).

**Conclusion:**

Our analysis failed to show cannabis to be protective against relapse to opioid use in patients receiving buprenorphine-naloxone for OUD. We identified that individuals who inject drugs, are on higher doses of buprenorphine-naloxone, or have been in treatment for less than two years have a higher hazard for relapse. The presence of such factors may thus warrant closer patient follow-up and more stringent treatment protocols to mitigate risk of relapse and potential overdose.

## Introduction

Opioid use disorder (OUD) has led to a serious public health crisis and epidemic. In the United States, drug overdoses remain the leading cause of death in those under 45 years of age (1), with opioid overdoses being the main driver of fatalities (2, 3). Unfortunately, studies have shown that more than 90% of opioid overdose-related deaths are unintentional (4). Opioid agonist therapy (OAT), by means of methadone and buprenorphine-naloxone, are the mainstay for pharmacological treatment of OUD (5, 6). The latter has become increasingly favored due to its comparable effectiveness but safer side effect profile and much lower risk of misuse and overdose (5). Despite the magnitude of the opioid crisis, less than 35% of patients with OUD seek treatment, of whom less than one third actually remain in treatment as intended due to high rates of relapse and loss to follow-up (7–9).

Few studies have aimed to identify predictors of relapse amongst patients receiving buprenorphine-naloxone therapy as a primary outcome. These studies have been limited by their retrospective design, smaller sample sizes, and statistical methods challenges (8, 10–12). We aim to conduct a survival analysis, using time-to-event data, to analyze predictors of relapse amongst patients receiving buprenorphine-naloxone for OUD. Although clinical data are still lacking, there is emerging but conflicting evidence that cannabis use may mitigate opioid use, possibly through triggering endogenous opioid release and amplifying the analgesic effect of opioids (13–17). We are, therefore, particularly interested in identifying the association between cannabis use and time to relapse amongst this population. Our group recently published a manuscript identifying that daily cannabis use is associated with a lower likelihood of continued opioid use during OAT treatment, amongst patients on both methadone and buprenorphine-naloxone (18). This study focuses on identifying predictors of relapse amongst patients who are abstinent at study onset, and focuses on the subpopulation of patients receiving buprenorphine-naloxone. We hypothesize that cannabis use is protective for relapse into opioid use in patients using cannabis during OAT treatment, due to emerging evidence about its potential benefits at mitigating withdrawal amongst patients with OUD (13–15, 18).

### Research question

What is the association between cannabis use and relapse amongst patients receiving buprenorphine-naloxone for OUD?

## Materials and methods

### Study design

We conducted our analyses using data collected from an ongoing longitudinal study entitled Pharmacogenetics of Opioid Substitution Treatment Response (POST) (19). This is a prospective cohort study aimed at assessing the association between biopsychosocial factors and opioid agonist therapy (OAT) outcomes. Data for the study were collected from 31 clinical sites across Ontario, Canada, between May 2018 and October 2020. The protocol for this study has previously been described (19). The study has been approved by the Hamilton Integrated Research Ethics Board (#4556) and funded by the Canadian Institute for Health Research (CIHR). The current study is reported according to the Strengthening the Reporting of Observational Studies in Epidemiology (STROBE) statement (20).

In order to be included into the present study, participants had to be at least 16 years of age or older, have provided written informed consent, be receiving buprenorphine-naloxone therapy for OUD, and have a urine toxicology screen negative for illicit opioids at the time of study entry. OUD is defined as per the Diagnostic and Statistical Manual of Mental Disorders, 5th Edition (DSM-5) (21). All participants underwent a semi-structured baseline interview with trained research staff whereby baseline demographic information, past medical and substance use histories were obtained by self-report. Frequency, compound of choice, amount, and route of cannabis and illicit benzodiazepine use in the past 30 days were ascertained by self-report using the Maudsley Addiction Profile (22). We included illicit benzodiazepines use as have previously shown it to be a predictor of accelerated relapse amongst patients with OUD on methadone maintenance therapy (23). As part of the usual treatment for OUD, participants underwent regular urine toxicology screens, typically on a weekly to bi-weekly basis. The FaStep Assay (Trimedic Supply Network Ltd, Concord, ON, Canada) was used to detect morphine, oxycodone, fentanyl, methadone metabolite, and buprenorphine, as well as other non-opioid substances (19). Participants were followed at 3 months intervals, for up to 12 months. At study entry and each follow-up, the following data were obtained from participants’ electronic medical records: current buprenorphine-naloxone dose, length of time on treatment, date of last dose taken, and results of all urine toxicology screens within the preceding three months period.

### Statistical analysis

Analyses were conducted using STATA version 13.0 (24). We used descriptive statistics to summarize participants’ baseline characteristics. Continuous variables were expressed using mean and standard deviation, whereas categorical variables were expressed using percentages. We employed two-sample *t*-tests (for continuous variables) and Pearson’s chi-square tests (for categorical variables) to compare baseline participants’ characteristics between relapsing and non-relapsing participants. We used Kaplan–Meier curves to estimate time to relapse for cannabis users and non-users. We compared the survival times between by cannabis use using the log-rank method. We then employed a multivariable Cox-proportional hazard model to assess the association between time to relapse and cannabis use, while adjusting for clinically important variables that may impact treatment outcomes. Specifically, we adjusted our model for age (continuous variable), duration of time in treatment (categorical variable), current dose (continuous variable), marital status (dichotomous variable), employment status (dichotomous variable), illicit benzodiazepine use (dichotomous variable), and history of injection drug use (dichotomous variable). Given that the continuous variable time in treatment violated the proportional hazard assumption, it was converted to a categorical variable which satisfied the assumption. We chose cut-off points of less than or equal to 12 months (*n* = 87), 12–24 months (*n* = 146), 24–36 months (*n* = 90), and greater than 36 months (*n* = 143). The cut-off points were chosen based on clinically important time points, while also ensuring that a sufficient number of participants remained in each of the categories. The minimum recommended treatment duration is 12 months, and this was used as the initial cut-off, followed by each additional year, as longer duration in treatment is an indicator of stability (5). We used time of entry into the study as the time origin, and time in study (days) as the time scale. We defined time to relapse as the time from study enrolment to the time of first urine toxicology screen positive for a non-prescribed opioid. We conducted identical analyses within the cannabis users, assessing the association between daily cannabis use and time to relapse, compared to non-daily use. We assessed for multi-collinearity by calculating the variance inflated factor (VIF), and considered a VIF of greater or equal to five or ten to suggest moderate or severe multi-collinearity, respectively. We followed the general rule of thumb of 10 events per variable for achieving adequate power in a cox model (25).

### Handling of censored data

At each follow-up, data regarding the reason censored participants may no longer be in treatment were recorded, as well as the date of their last urine toxicology screen and date of the last buprenorphine-naloxone dose consumed. If censored data were deemed to be random, independent and non-informative based on our assessment, then basic Kaplan–Meier plots and Cox-proportional hazard functions were used to handle censored data (26, 27). If, based on the reason for censoring, it was deemed that censoring may have been informative, then a worst-case imputation approach was used as a sensitivity analysis to assess the robustness of the findings (26, 27).

## Results

### Participant characteristics

**FIGURE 1:**
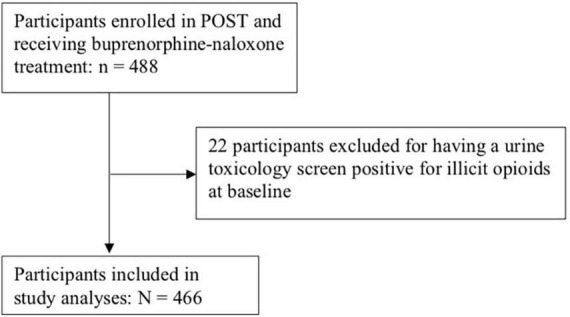
Participant-flow diagram.

Data from 466 participants receiving buprenorphine-naloxone therapy were available for analysis. Please see Figure 1 for participant flow diagram. Participants were followed between May 2018 and October 2020, for a median 165 days [interquartile range (IQR): 37, 357], and a total of 85,451 person-years of follow-up. Forty-six percent of participants relapsed during the one year study period, constituting an event rate of 0.25 events per 100 person-years. Of the 254 participants with no documented relapse episodes, 148 participants completed 12-month follow-up without a relapse (31.8% of the total study sample).

The mean age of participants was 39 years, and approximately half (46%) were female. The average dose of buprenorphine-naloxone was 16.7 mg [standard deviation (SD) 16.8] in the group that relapsed, compared to 10.8 mg (SD 8.70) in the group that did not relapse (*p* < 0.001). A larger proportion of those who relapsed (17%) endorsed injection drug use, compared to those who did not relapse (5%) during follow-up (*p* < 0.001). Please see Table 1 for complete baseline patient characteristics.

**TABLE 1:** Baseline participant characteristics.

Participant characteristic	Total (*n* = 466)	Relapsed (*n* = 212)	Not relapsed (i.e., censored, *n* = 254)	*P*-value
**Mean (SD)**				
Age (years)	38.59 (10.73)	38.32 (10.44)	38.82 (10.99)	0.620
Time on treatment (months)	34.94 (33.32)	34.76 (37.22)	35.10 (31.77)	0.916
Buprenorphine dose (milligrams)	13.48 (13.35)	16.71 (16.80)	10.79 (8.71)	<0.001
***N* (%)**				
Female	215 (46.14)	98 (46.23)	117 (46.06)	0.972
Cannabis user	225 (48.28)	107 (50.47)	118 (46.47)	0.388
Married	144 (30.90)	65 (30.66)	79 (31.10)	0.918
Employed	185 (39.70)	74 (34.91)	111 (43.70)	0.053
Injection drug use	49 (10.52)	37 (17.45)	12 (4.72)	<0.001
Illicit benzodiazepine use	29 (6.22)	17 (8.01)	12 (4.72)	0.143

### Primary analyses

#### Multivariable-adjusted Cox regression: Predictors of relapse and the association with cannabis use

In the multivariable-adjusted Cox regression, we found that cannabis use was not protective against relapse [hazard ratio (HR) = 1.03, 95% confidence interval (CI): 0.78, 1.36, *p* = 0.84]. We found that participants who have been in treatment between two and three years had a 44% decrease in the hazard of relapse compared to those in treatment for less than a year (HR = 0.56, 95% CI: 0.34, 0.92, *p* = 0.021). Similarly, those in treatment for three or more years had a 37% reduction in the hazard of relapse compared to those in treatment for less than a year (HR = 0.63, 95% CI: 0.40, 0.98, *p* = 0.041). We also found that the hazard of relapse was 2.6 times higher for participants who injected drugs compared to those who did not (HR = 2.61, 95% CI: 1.74, 3.91, *p* < 0.01). Finally, we find that for every 1 or 10 mg increase in the participants’ buprenorphine-naloxone dose, the hazard of relapse is 2 or 22% greater, respectively (HR = 1.02 per 1 mg increase in dose, 95% CI: 1.01, 1.03, *p* < 0.001). The VIF of included variables ranged between 1.02 and 2.04, thus ruling out multi-collinearity. See Table 2. The results were unchanged in a sensitivity analysis conducted within cannabis users, assessing the association between daily cannabis use and time to relapse, compared to non-daily use, while adjusting for the same covariates [data not shown].

**TABLE 2:** Multivariable Cox regression analysis: Predictors of relapse amongst patients receiving buprenorphine-naloxone for OUD (*N* = 466).

Variable	Hazard ratio	95% CI	*P*-value
Cannabis use	1.03	0.78, 1.36	0.835
Female	0.89	0.67, 1.19	0.431
Age (years)	1.00^†^	0.98, 1.01	0.697
Currently employed	0.76	0.57, 1.02	0.069
Married	1.01	092, 1.10	0.896
Injection drug use	2.61	1.74, 3.91	<0.001
Amt. of last buprenorphine-naloxone dose (milligrams)	1.02^†‡^	1.01, 1.03	<0.001
Illicit benzodiazepine use	1.42	0.83, 2.41	0.200
Time on treatment*			
- > 12 months and ≤ 24 months	1.04	0.69, 1.58	0.837
- > 24 months and ≤ 36 months	0.56	0.34, 0.92	0.021
- > 36 months	0.63	0.40, 0.98	0.041

^†^Hazard ratio calculated per one unit change of independent variable.

^‡^HR = 1.22 per 10 mg increase in buprenorphine-naloxone dose.

*Compared to ≤12 months.

#### Kaplan–Meier estimates: Association between cannabis use and relapse

**FIGURE 2:**
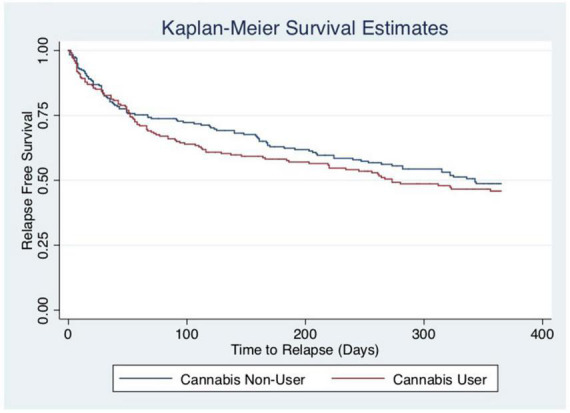
Kaplan–Meier curves by current cannabis use. The y-axis and x-axis labels are probability of survival and time until relapse (days), respectively. The log-rank test reveals that the survival distribution between those who currently use cannabis and those who do not is not statistically different (chi-square: 0.77, *p* = 0.3804).

Unadjusted Kaplan–Meier curves reveal that cannabis users have a trend towards shorter time to relapse, but that this association is not statistically significant (*p* = 0.380). Please see Figure 2. The log-rank test remained statistically non-significant in a sensitivity analysis amongst cannabis users, assessing association between daily cannabis use and relapse, compared to non-daily cannabis use [data not shown]. This is consistent with the findings of the multivariable-adjusted Cox model above.

### Sensitivity analysis: Handling of censored data

Of the 254 (55%) with no documented relapse episodes, 245 were right censored and 9 were interval censored. Of the 245 participants who were right censored, 148 were censored due to completing the 12 months follow-up (study end), 18 were transferred to another provider, 11 completed treatment and were discharged from the clinic, and 8 were incarcerated. We consider these participants to be censored for non-informative reasons. The remaining 60 participants who were right censored, and 9 who were interval censored, were lost to follow-up as they stopped attending their clinic appointments. It may be argued that these participants who are lost to follow-up have relapsed, and as such, we conducted a sensitivity analysis whereby we assumed that all 69 participants who were censored for having withdrawn from treatment had relapsed at the time of censoring. The association between cannabis use and time to relapse remained non-significant in this multivariable-adjusted Cox regression analysis (HR = 0.96, 95% CI: 0.76, 1.22, *p* = 0.755). The log-rank test comparing unadjusted survival times stratified by sex also indicated no statistically significant difference in the time to relapse between cannabis users and non-users (*p* = 0.557). The association between time-to-relapse and the remainder of the predictors assessed remained unchanged from the primary analysis, with the exception of employment status whereby those who were employed had a 25% reduction in the hazard of relapse compared to those who were unemployed (HR = 0.76, 95% CI 0.58, 0.98, *p* = 0.031).

## Discussion

Our study identifies several predictors of opioid relapse for patients receiving buprenorphine-naloxone therapy, one of the first-line agents for OUD. While relapse in any substance use disorder is an important outcome, it is particularly relevant in OUD wherein patients lose tolerance to opioids within days of stopping use and are at significantly heightened risk of overdose with relapse to smaller amounts of opioids. Although buprenorphine is known to have affinity for the mu-opioid receptors and therefore helps maintain one’s tolerance to opioids, the level of tolerance depends on the plasma concentration level and it is not known how this equates to tolerance to fentanyl—a synthetic opioid with much higher potency (28). Identifying patients at higher risk of relapse is therefore an integral aspect of harm reduction for managing patients with OUD, as we know that over 90% of opioid overdose deaths are unintentional (4, 29). In our study, we find that participants who inject drugs or are on a higher dose of buprenorphine-naloxone have a significantly higher hazard of relapse at any point in time, whereas being in treatment for more than two years is associated with a lower hazard of relapse. Our findings also indicate that cannabis use does not have a significant association with relapse to opioid use and we could not show protective effect of cannabis in this study amongst participants receiving buprenorphine-naloxone for OUD, even after adjusting for other clinically important variables.

We find that participants who inject drugs and those who are in treatment for a shorter period of time have a higher hazard of relapse at any point in time. This is likely explained by the fact that opioids have higher bioavailability when injected intravenously and intravenous use is typically an indicator of more severe OUD as well as poorer outcome (23). Similarly, the longer one is in treatment, the more stable they are likely to be. Thus it is expected that individuals who are in treatment for a shorter period of time would be more likely to relapse (5, 10). Lastly, individuals with more severe OUD, including those who inject drugs, often require higher doses of buprenorphine-naloxone. As such, it once again seems plausible that the individuals with higher doses had a higher hazard of relapse due to them having a more severe OUD, necessitating the higher dose of treatment in the first place. This is consistent with prior research (5, 10).

Our findings add to the available literature investigating the association between cannabis use and OUD. Emerging evidence suggests that cannabis may serve as a harm reduction strategy to mitigate opioid consumption, as the active component delta-9-tetrahydocannabinol (THC) may amplify the analgesic effects of consumed opioids as well as trigger endogenous opioid release (30–33). However, there is substantial heterogeneity in the evidence to support this association or mechanism of action (13–15). Similar to our findings, a cross-sectional analysis of 777 patients receiving methadone maintenance therapy for OUD found that cannabis use was not associated with illicit opioid use during treatment (14).

The study results may be impacted by the missing data on a number of individuals. As discussed above, 254 individuals were censored, of which 69 could have been informative censoring as they dropped out of treatment at some point during follow-up. In order to address this, we used the worst-case scenario imputation method, whereby we assumed that these 69 individuals relapsed at the time of drop out. This analysis yielded a similar finding, that cannabis use is not protective against relapse to opioids, highlighting the robustness of our findings.

Our findings are strengthened by the fact that all individuals who were censored were followed up and the timing as well as reason for censoring were documented. This allowed us to more reliably make a judgment regarding informative censoring, so as to conduct the appropriate analyses discussed above. Another strength of our study is that our outcome, time to relapse, is objective on the basis of a positive urine toxicology screen, and that it is collected on a weekly to biweekly basis, providing a relatively accurate timing of relapse. One limitation of this study is that it is certainly possible for an individual to have relapsed prior to enrolment into the study. These individuals are not necessarily excluded, or left truncated, however, as long as their urine toxicology screen at study enrollment was negative for illicit opioids. Given we are interested in time-to-relapse, individuals who are actively using illicit opioids while on OAT are not part of our study population. It is not possible for us to know whether these individuals had ever achieved a period of sobriety and then relapsed (thus left truncated), or never achieved a period of sobriety to begin with (thus not part of our target study population). Nonetheless, only 22 participants had a urine toxicology screen that was positive for illicit opioids at baseline, of which only a fraction represent true relapses, thus would be unlikely to have biased our results (see Figure 1). Lastly, another limitation is the fact that time origin for this study is time of study enrolment, whereas patients could have been receiving treatment for varying periods of time. We have attempted to mitigate this by adjusting our model for length of time on treatment.

Taken together, our study identifies that there is neither a positive nor protective association between cannabis use and time-to-relapse among patients receiving buprenorphine-naloxone for OUD. The majority of research evaluating cannabis use and outcomes of patients receiving opioid agonist therapy has focused on illicit opioid use and retention in treatment as study outcomes. This is the first study, to our knowledge, to investigate its impact on time-to-relapse. Relapse is an important outcome due to the serious implications associated with loss of tolerance and risk of overdose, as well as the fact that abstinence from opioid use is what patients consider to be the most important outcome of treatment for OUD (34). Our study calls upon further research to investigate the association between cannabis use and opioid use so as to optimize treatment outcomes, especially as the prevalence of cannabis use continues to rise (35). Moreover, we identified that patients who inject drugs, are on higher doses of buprenorphine-naloxone, or have been in treatment for less time have a higher hazard of relapse. More stringent monitoring during treatment may be warranted to mitigate relapse risk amongst these patients, and future research is needed to further investigate these associations and replicate our findings.

## Conclusion

We found that cannabis use was not protective against relapse to opioid use in patients receiving buprenorphine-naloxone for OUD. We identified that individuals who inject drugs drug users, are on higher doses of buprenorphine-naloxone, or have been in treatment for less than 2 years have a higher hazard for relapse. The presence of such factors may thus warrant closer patient follow-up and more stringent treatment protocols to mitigate risk of relapse and potential overdose. Future research aimed at delineating the potential protective or negative consequences cannabis use may have on treatment outcomes for patients with OUD is recommended.

## Data availability statement

The raw data supporting the conclusions of this article will be made available by the authors, without undue reservation.

## Ethics statement

The studies involving human participants were reviewed and approved by Hamilton Integrated Research Ethics Board. The participants provided their written informed consent to participate in this study.

## Author contributions

LN and ZS conceived the research question and protocol. NS, AH, and JH collected participant data for the study. LN, NS, and JH formatted and extracted the relevant data for the study. LN, LT, and ZS conducted the study analyses. All authors contributed equally to the writing and revision of the manuscript and approved the final version of the manuscript.
